# In Depth Analysis of the *Helicobacter pylori cag* Pathogenicity Island Transcriptional Responses

**DOI:** 10.1371/journal.pone.0098416

**Published:** 2014-06-03

**Authors:** Andrea Vannini, Davide Roncarati, Marco Spinsanti, Vincenzo Scarlato, Alberto Danielli

**Affiliations:** Department of Pharmacy and Biotechnology (FaBiT), University of Bologna, Bologna, Italy; Institut Pasteur Paris, France

## Abstract

The severity of symptoms elicited by the widespread human pathogen *Helicobacter pylori* is strongly influenced by the genetic diversity of the infecting strain. Among the most important pathogen factors that carry an increased risk for gastric cancer are specific genotypes of the *cag* pathogenicity island (*cag*-PAI), encoding a type IV secretion system (T4SS) responsible for the translocation of the CagA effector oncoprotein. To date, little is known about the regulatory events important for the expression of a functional *cag*-T4SS. Here we demonstrate that the *cag*-PAI cistrons are subjected to a complex network of direct and indirect transcriptional regulations. We show that promoters of *cag* operons encoding structural T4SS components display homogeneous transcript levels, while promoters of *cag* operons encoding accessory factors vary considerably in their basal transcription levels and responses. Most *cag* promoters are transcriptionally responsive to growth-phase, pH and other stress-factors, although in many cases in a pleiotropic fashion. Interestingly, transcription from the P*cagζ* promoter controlling the expression of transglycolase and T4SS stabilizing factors, is triggered by co-culture with a gastric cell line, providing an explanation for the increased formation of the secretion system observed upon bacterial contact with host cells. Finally, we demonstrate that the highly transcribed *cagA* oncogene is repressed by iron limitation through a direct *apo*-Fur regulation mechanism. Together the results shed light on regulatory aspects of the *cag*-PAI, which may be involved in relevant molecular and etiological aspects of *H. pylori* pathogenesis.

## Introduction

*Helicobacter pylori* is a major human pathogen that colonizes the gastric epithelium of more than half of humankind, worldwide. Etiology of *H. pylori* infections ranges from mild to acute symptoms, including gastric inflammations and duodenal diseases. While symptomatic infections can be successfully treated with antimicrobial drugs, the untreated and asymptomatic infections persist over decades, promoting the long term chronic inflammation and insurgence of peptic ulcers and gastric cancer [1], [2].

The severity of symptoms largely depends on the genetic diversity of the infecting strain [2], and particularly on specific genotypes of virulence-associated genes, such as the *cag* pathogenicity island (*cag*-PAI). The latter is a 38 kb multi-operon locus coding for 28 putative ORFs, six of which were identified as homologues of the basic type IV secretion system (T4SS) represented by the *Agrobacterium tumefaciens* virB operon [3]. The *H. pylori cag*-T4SS promotes injection of the CagA bacterial oncoprotein into host cells [4], as well as a CagA-independent induction of interleukin-8 secretion via the host AP-1 and NF-kB signaling pathways [5]. Notably, chemokine induction requires direct contact of the bacteria with epithelial host cells [6]. Host integrin receptors are engaged by the CagL protein to promote CagA translocation across the host cell membrane and to activate the Src tyrosine kinase for CagA phosphorylation [7], resulting in cytoskeletal rearrangements of the gastric epithelium [8]. Seventeen genes of the *cag*-PAI are essential for CagA translocation, including three genes HP0524 (virD homologue), HP0526, and HP0540 that are dispensable for IL-8 induction [9]. Moreover, several genes of the *cag*-PAI are not necessary for either CagA translocation or IL-8 induction, and their role in Cag-T4SS assembly and virulence remains to be elucidated [10], [11].

Proper stoichiometric ratio of gene products and appropriate expression in time and space in the host allows the assembly of functional secretion systems [12]. Polarity effects modulating transcription and translation can contribute to this regulation, but in many bacteria the assembly requires finely tuned regulatory mechanisms. For example, it has been shown that dedicated transcriptional regulators are frequently encompassed within the PAIs of type III and type IV secretion systems. Transcriptional regulators such as Fur, HilA, HilC, HilD, InvF and ExsA, controlling *Salmonella enterica* pathogenicity island (SPI1) and *Pseudomonas aeruginosa* T3SS gene expression, respectively, are informative examples to date [13]–[15].

*H. pylori* is unusual in this respect. In fact, despite multiple operons with oppositely phased ORFs and frequent intergenic regions spanning >70 bp, which advocate the existence of multiple *cag* promoters, the *cag*-PAI lacks genes with annotated regulatory function [16]. In the past, only one promoter region of the divergent *cagAB* genes has been analyzed in detail [17]. In addition, it has also been shown that some of the *H. pylori cag* genes may be responsive to acid pH [18]–[20], or free iron [21]–[24],while others may be induced upon contact with the host cells [25]. However, little is known about the regulatory events behind these processes and the regulators that transduce these signals are not known.

As the expression of a functional *cag*-T4SS is intimately linked to the etiology of *H. pylori*, a systematic study of *cag* gene transcription will provide insights into timing and regulation of *H. pylori* virulence. Here we functionally characterize the main *cag* promoters and their transcriptional responses after different stress signals, demonstrating a direct regulatory role of *apo*-Fur on *cagA* and a consistent transcriptional induction of the *cagζεδγ* cistron upon *H. pylori* interaction with host cells.

## Materials and Methods

### Bacterial strains and growth conditions

All *H. pylori* strains used are listed in Table 1. Bacteria were recovered from −80°C glycerol stocks and propagated on BBLBrucella (BD) agar plates containing 5% fetal calf serum (Oxoid), 0.2% cyclodextrin, and Dent's or Skirrow's antibiotic supplement. Cultures were grown for 24–48 hours at 37°C in a water-jacketed thermal incubator (9% CO_2_, 91% air atmosphere, and 95% humidity) or in jars using CampyGen (Oxoid) gas-packs. Liquid cultures were grown in BBL Brucella Broth supplemented with 5% fetal calf serum and Dent's or Skirrow's antibiotic supplement at 37°C with gentle agitation (125 rpm), in glass or tissue-culture flasks with vented cap. When required, Brucella agar plates or liquid broth were supplemented with chloramphenicol (30 µg ml^−1^) and kanamycin (25 µg ml^−1^). *H. pylori* transformants were obtained by double homologous recombination of the naturally competent G27 strain using 5 µg of transforming DNA, as previously described [26]; positive clones were selected on Brucella agar plates supplemented with chloramphenicol, according to the resistance phenotype conferred by the *cat* cassette (Cm^R^). *E. coli* DH5α cultures for cloning purposes were grown in Luria-Bertani broth. Ampicillin (100 µg ml^−1^), chloramphenicol (30 µg ml^−1^) and kanamycin (25 µg ml^−1^) were added when required.

**Table 1:** Strains and plasmid used in this study.

Strain or Plasmid	Genotype or description	Source or reference
*E. coli* strains		
DH5α	supE44 ΔlacU169 (Φ80 lacZΔM15) hsdR17 recA1 endA1 gyrA96 thi-1 relA1β	
*H. pylori* strains		
G27	Clinical isolate; wild-type parental strain	
G27(*fur::km*)	G27 derivative; bp 25 to 434 of the *fur (HP1027)* coding sequence replaced by a *km* cassette; Km^R^	[26]
G27(*nikR::km*)	G27 derivative; bp 88 to 417 of the *nikR (HP1338)* coding sequence replaced by a *km* cassette; Km^R^	[29]
G27(*hspR::km*)	G27 derivative; bp 66 to 334 of the *hspR (HP1025)* coding sequence replaced by a *km* cassette; Km^R^	[39]
G27(*hrcA::km*)	G27 derivative; bp 156 to 375 of the *hrcA (HP0111)* coding sequence replaced by a *km* cassette; Km^R^	[39]
G27(*arsS::cat*)	G27 derivative; bp 3 to 1290 of the *arsS (HP0164-HP0165)* coding sequence replaced by a *cat* cassette; Cm^R^	This study
G27*lux*	G27 derivative carrying the *km* cassette and the promoterless *Photorhabdus luminescens luxCDABE* operon in the *vacA* locus; Km^R^	[27]
G27*lux* P*_cagζ_*	*vacA::cat-P_cagζ_luxCDABE*; G27*lux* derivative obtained by transformation and subsequent double homologous recombination with plasmid PVCC with plasmid PVCC::P*_cagζ_*; Cm^R^	This study
G27*lux* P*_cagζ_*-5'UTR	*vacA::cat-P_cagζ_-cagζ-5'UTR-luxCDABE*; G27*lux* derivative obtained by transformation with plasmid PVCC::P*_cagζ_*-5'UTR; Cm^R^	This study
G27*lux* P*_cagV_*	*vacA::cat-P_cagV_luxCDABE*; G27*lux* derivative obtained by transformation with plasmid PVCC::P*_cagV_*; Cm^R^	This study
G27*lux* P*_cagV _*-5'UTR	*vacA::cat-P_cagV_-cagV5'UTR-luxCDABE*; G27*lux* derivative obtained by transformation with plasmid PVCC::P*_cagV_*-5'UTR; Cm^R^	This study
G27*lux* P*_cagU_*	*vacA::cat-P_cagU_luxCDABE*; G27*lux* derivative obtained by transformation with plasmid PVCC::P*_cagU_*; Cm^R^	This study
G27*lux* P*_cagU_*-5'UTR	*vacA::cat-P_cagU_-cagU5'UTR-luxCDABE*; G27*lux* derivative obtained by transformation with plasmid PVCC::P*_cagU_*-5'UTR; Cm^R^	This study
G27*lux* P*_cagQ_*	*vacA::cat-P_cagQ_luxCDABE*; G27*lux* derivative obtained by transformation with plasmid PVCC::P*_cagQ_*; Cm^R^	This study
G27*lux* P*_cagQ_*-5'UTR	*vacA::cat-P_cagQ_-cagQ5'UTR-luxCDABE*; G27*lux* derivative obtained by transformation with plasmid PVCC::P*_cagQ_*-5'UTR; Cm^R^	This study
G27*lux* P*_cagS_*	*vacA::cat-P_cagS_luxCDABE*; G27*lux* derivative obtained by transformation with plasmid PVCC::P*_cagS_*; Cm^R^	This study
G27*lux* P*_cagS_*-5'UTR	*vacA::cat-P_cagS_-cagS5'UTR-luxCDABE*; G27*lux* derivative obtained by transformation with plasmid PVCC::P*_cagS_*-5'UTR; Cm^R^	This study
G27*lux* P*_cagP_*	*vacA::cat-P_cagP_luxCDABE*; G27*lux* derivative obtained by transformation with plasmid PVCC::P*_cagP_*; Cm^R^	This study
G27*lux* P*_cagP_*-5'UTR	*vacA::cat-P_cagP_-cagP5'UTR-luxCDABE*; G27*lux* derivative obtained by transformation with plasmid PVCC::P*_cagP_*-5'UTR; Cm^R^	This study
G27*lux* P*_cagM_*	*vacA::cat-P_cagM_luxCDABE*; G27*lux* derivative obtained by transformation with plasmid PVCC::P*_cagM_*; Cm^R^	This study
G27*lux* P*_cagM _*-5'UTR	*vacA::cat-P_cagM_-cagM5'UTR-luxCDABE*; G27*lux* derivative obtained by transformation with plasmid PVCC::P*_cagM_*-5'UTR; Cm^R^	This study
G27*lux* P*_cagF_*	*vacA::cat-P_cagF_luxCDABE*; G27*lux* derivative obtained by transformation with plasmid PVCC::P*_cagF_*; Cm^R^	This study
G27*lux* P*_cagF _*-5'UTR	*vacA::cat-P_cagF_-cagF5'UTR-luxCDABE*; G27*lux* derivative obtained by transformation with plasmid PVCC::P*_cagF_*-5'UTR; Cm^R^	This study
G27*lux* P*_cagC_*	*vacA::cat-P_cagC_luxCDABE*; G27*lux* derivative obtained by transformation with plasmid PVCC::P*_cagC_*; Cm^R^	This study
G27*lux* P*_cagC_*-5'UTR	*vacA::cat-P_cagC_-cagC5'UTR-luxCDABE*; G27*lux* derivative obtained by transformation with plasmid PVCC::P*_cagC_*-5'UTR; Cm^R^	This study
G27*lux* P*_cagB_*	*vacA::cat-P_cagB_luxCDABE*; G27*lux* derivative obtained by transformation with plasmid PVCC::P*_cagB_*; Cm^R^	This study
G27*lux* P*_cagB _*-5'UTR	*vacA::cat-P_cagB_-cagB5'UTR-luxCDABE*; G27*lux* derivative obtained by transformation with plasmid PVCC::P*_cagB_*-5'UTR; Cm^R^	This study
G27*lux* P*_cagA_*	*vacA::cat-P_cagA_luxCDABE*; G27*lux* derivative obtained by transformation with plasmid PVCC::P*_cagA_*; Cm^R^	This study
G27*lux* P*_cagA_*-5'UTR	*vacA::cat-P_cagA_-cagA5'UTR-luxCDABE*; G27*lux* derivative obtained by transformation with plasmid PVCC::P*_cagA_*-5'UTR; Cm^R^	This study
Plasmids		
pBluescript KS II	Cloning vector, Ap^R^	Stratagene
pGEM-T Easy	Cloning vector, Ap^R^	Promega
pBS::*cat*	pBluescript KS II derivative carrying a *Campylobacter coli cat* cassette; Ap^R^, Cm^R^	[27]
pBS::Δ*arsS*	pBluescript KS II derivative, carrying a 460 bp XbaI-BglII fragment amplified on chromosomal DNA of *H. pylori* with oligos 163f_Xba and 163r_Bgl, BglI/BamHI *cat* cassette and a 616 bp BglII-HindIII fragment amplified with 166f_Bgl and 166r_Hin; Ap^R^, Cp^R^	This study
pGEM-P*_cagAB_*	pGEM-T Easy derivative, carrying a 403 bp fragment amplified on chromosomal DNA of *H. pylori* with oligos Lux547R and Lux546F, encompassing P*_cagA_*-P*_cagB_* promoter regions; Ap^R^, Cp^R^	This study
pVCC	Suicide transformation vector for promoter-*lux* fusions; Ap^R^, Cm^R^	[27]
PVCC::P*_cagζ_*	pVCC derivative carrying a 122 bp BamHI/BglII fragment amplified on chromosomal DNA of *H. pylori* with oligos Lux519F and Lux520RS, encompassing 115 bp of the P*_cagζ_* promoter and the first 7 bp of the *cagζ* 5'UTR cloned upstream of *luxC*; Ap^R^, Cm^R^	This study
PVCC::P*_cagζ_*-5'UTR	pVCC derivative carrying a 166 bp BamHI/BglII fragment from Lux519F and Lux520RL oligos, encompassing 115 bp of the P*_cagζ_* promoter and the *cagζ* 5'UTR (51 bp) cloned upstream of *luxC*; Ap^R^, Cm^R^	This study
PVCC::P*_cagV_*	pVCC derivative carrying a 306 bp BamHI/BglII fragment from VS530FS and VS531RL oligos, encompassing 300 bp of the P*_cagV_* promoter and the first 6 bp of the cagV 5'UTR cloned upstream of *luxC*; Ap^R^, Cm^R^	This study
PVCC::P*_cagV_*-5'UTR	pVCC derivative carrying a 366 bp BamHI/BglII fragment from VS530FL and VS531RS oligos, encompassing 269 bp of the P*_cagV_* promoter and the *cagV* 5'UTR (97 bp) cloned upstream of *luxC*; Ap^R^, Cm^R^	This study
PVCC::P*_cagU_*	pVCC derivative carrying a 366 bp BamHI/BglII fragment from VS530FL and VS531RS oligos, encompassing 361 bp of the P*_cagU_* promoter and the first 5 bp of the *cagU* 5'UTR cloned upstream of *luxC*; Ap^R^, Cm^R^	This study
PVCC::P*_cagU_*-5'UTR	pVCC derivative carrying a 306 bp BamHI/BglII fragment from VS530FS and VS531RL oligos, encompassing 270 bp of the P*_cagU_* promoter with the *cagU* 5'UTR (35 bp) cloned upstream of *luxC*; Ap^R^, Cm^R^	This study
PVCC::P*_cagS_*	pVCC derivative carrying a 180 bp BamHI/BglII fragment from VS534FS and VS534R2 oligos, encompassing 177 bp of the P*_cagS_* promoter and the first 3 bp of the *cagS* 5'UTR cloned upstream of *luxC*; Ap^R^, Cm^R^	This study
PVCC::P*_cagS_*-5'UTR	pVCC derivative carrying a 241 bp BamHI/BglII fragment from VS534F and VS534R2 oligos, encompassing 177 bp of the P*_cagS_* promoter and the *cagS* 5'UTR (64 bp) cloned upstream of *luxC*; Ap^R^, Cm^R^	This study
PVCC::P*_cagQ_*	pVCC derivative carrying a 304 bp BamHI/BglII fragment from VS535FS and VS535R oligos, encompassing 301 bp of the P*_cagQ_* promoter and the first 3 bp of the *cagQ* 5'UTR cloned upstream of *luxC*; Ap^R^, Cm^R^	This study
PVCC::P*_cagQ_*-5'UTR	pVCC derivative carrying a 439 bp BamHI/BglII fragment from VS535F and VS535R oligos, encompassing 301 bp of the P*_cagQ_* promoter and the *cagQ* 5'UTR (138 bp) cloned upstream of *luxC*; Ap^R^, Cm^R^	This study
PVCC::P*_cagP_*	pVCC derivative carrying a 204 bp BamHI/BglII fragment from VS536FS and VS537RL oligos, encompassing 201 bp of the P*_cagP_* promoter and the first 3 bp of the *cagP* 5'UTR cloned upstream of *luxC*; Ap^R^, Cm^R^	This study
PVCC::P*_cagP_*-5'UTR	pVCC derivative carrying a 394 bp BamHI/BglII fragment from VS536FL and Lux537R2 oligos, encompassing 170 bp of the P*_cagP_* promoter and the *cagP* 5'UTR (224 bp) cloned upstream of *luxC*; Ap^R^, Cm^R^	This study
PVCC::P*_cagM_*	pVCC derivative carrying a 394 bp BamHI/BglII fragment from VS536FL and Lux537R2 oligos, encompassing 388 bp of the P*_cagM_* promoter and the first 6 bp of the *cagM* 5'UTR cloned upstream of *luxC*; Ap^R^, Cm^R^	This study
PVCC::P*_cagM _*-5'UTR	pVCC derivative carrying a 204 bp BamHI/BglII fragment from VS536FS and VS537RL oligos, encompassing 167 bp of the P*_cagM_* promoter and the *cagM* 5'UTR (37 bp) cloned upstream of *luxC*; Ap^R^, Cm^R^	This study
PVCC::P*_cagF_*	pVCC derivative carrying a 308 bp BamHI/BglII fragment from VS543FS and VS543R oligos, encompassing 305 bp of the P*_cagF_* promoter and the first 3 bp of the *cagF* 5'UTR cloned upstream of *luxC*; Ap^R^, Cm^R^	This study
PVCC::P*_cagF_* -5'UTR	pVCC derivative carrying a 352 bp BamHI/BglII fragment from VS543F and VS543R oligos, encompassing 305 bp of the P*_cagF_* promoter and the *cagF* 5'UTR (47 bp) cloned upstream of *luxC*; Ap^R^, Cm^R^	This study
PVCC::P*_cagC_*	pVCC derivative carrying a 276 bp BamHI/BamHI fragment VS546FS and VS546R oligos, encompassing 274 bp of the P*_cagC_* promoter and the first 2 bp of the *cagC* 5'UTR cloned upstream of *luxC*; Ap^R^, Cm^R^	This study
PVCC::P*_cagC _*-5'UTR	pVCC derivative carrying a 300 bp BamHI/BamHI fragment from VS546F and VS546R oligos, encompassing 274 bp of the P*_cagC_* promoter and the *cagC* 5'UTR (26 bp) cloned upstream of *luxC*; Ap^R^, Cm^R^	This study
PVCC::P*_cagB_*	pVCC derivative carrying a 261 bp BamHI/BglII fragment from VSorfxFS and VS547RL oligos, encompassing 257 bp of the P*_cagB_* promoter and the first 4 bp of the *cagB* 5'UTR cloned upstream of *luxC*; Ap^R^, Cm^R^	This study
PVCC::P*_cagB _*-5'UTR	pVCC derivative carrying a 324 bp BamHI/BglII fragment from VSorfxFL and VS547RS oligos, encompassing 155 bp of the P*_cagB_* promoter and the *cagB* 5'UTR (169 bp) cloned upstream of *luxC*; Ap^R^, Cm^R^	This study
PVCC::P*_cagA_*	pVCC derivative carrying a 324 bp BamHI/BglII fragment from VSorfxFL and VS547RS oligos, encompassing 321 bp of the P*_cagA_* promoter and the first 3 bp of the *cagA* 5'UTR cloned upstream of *luxC*; Ap^R^, Cm^R^	This study
PVCC::P*_cagA_*-5'UTR	pVCC derivative carrying a 261 bp BamHI/BglII fragment from VSorfxFS and VS547RL oligos, encompassing 156 bp of the P*_cagA_* promoter and the *cagA* 5'UTR (105 bp) cloned upstream of *luxC*; Ap^R^, Cm^R^	This study

### DNA manipulations

DNA amplification, restriction digests and ligations were all carried out with standard molecular techniques, with enzymes purchased from New England Biolabs. Large preparations of plasmid DNA were carried out with a NucleoBond Xtra Midi plasmid purification kit (Macherey-Nagel). DNA fragments for cloning purposes were extracted and purified using Qiaquick Kits (Qiagen, Inc.)

### Construction of an *arsS^-^* mutant

The *H. pylori* G27-derivative *arsS* knock-out mutant was obtained replacing the *arsS* gene (ORF HP0164HP0165) from position 3 to position 1290 of the coding sequence with a *Campylobacter coli cat* chloramphenicol resistance cassette (*cat*) by double homologous recombination using the pBS::Δ*arsS* suicidal vector. Primers 163f_Xba and 163r_Bgl (Table 2) were used to amplify and clone a 460 bp XbaI-BglII fragment encompassing the region upstream of *arsS*, corresponding to 359 bp of the 5′ region of the HP0163 open reading frame (ORF), 9 bp of the intergenic region and the 57 bp of the 3′ region of HP0164. Primers 166f_Bgl and 166r_Hin were used for amplification and cloning of the *arsS* downstream region: a 616 bp BglII-HindIII fragment carrying 585 bp of the 3′ region of the HP0166 ORF and 25 bp of the intergenic region upstream the HP0165 ORF. The *cat* cassette derived as BglII-BamHI fragment from pBS::*cat* was inserted between these two fragments and the final construct pBS::Δ*arsS* was used to transform *H. pylori*. The chloramphenicol-selected mutant strains were confirmed by PCR.

**Table 2:** Primers used for cloning of the promoter regions and for primer extension reactions.

Name	Sequence (5'-3')^a^	Source	Restriction site
520pe2	CTAATGAATCATAACGCTTGTC	This study	-
530pe1	ACCAAATTTTCATCAATCAAG	This study	-
531pe4	CATGATGCTCTGTTGTATC	This study	-
534pe3	GTTTTCGCATGTTATTACTC	This study	-
535pe1	ATAAGTAGCCACCAATCGCAAAC	This study	-
536pe17	AACGATTTGTTTGTTTATGC	This study	-
537pe8	CTCCAAACGCAACCAATGAG	This study	-
543pe3	GTTCACGCAAATTTTGTTTC	This study	-
546pe1	ACAACTTTCTTGTAGCTGTC	This study	-
OrfX	GCAACTCCATAGACCACTAAAG	[17]	-
cagN	GTCAATGGTTTCGTTAGTC	[17]	-
163f_Xba	GCCCATGGTCGGTGTCTAGACAAAAACACAAATCCGC	This study	Xba
163r_Bgl	GAAAATTTGAGATCTGTGAGCGGAGTGAAGGG	This study	BglII
166f_Bgl	CTTAAAAAAGATAGAGAGATCTAAAACCCCTTAACTC	This study	BglII
166r_Hin	CATGTAACCAAGCTTGATGAGCCATATACCGGC	This study	HindIII
Lux519F	TATAAGATCTAGTCCTTTTACAATTTGAGC	This study	BglII
VS520RS	ACTAGGATCCAAATTCATGTCATTATAGC	This study	BamHI
VS520RL	TGTGGGATCCATAGTGTTACCTCCATAAG	This study	BamHI
VS530FS	CTTAGGATCCTTTCAGTTATAGTATAG	This study	BamHI
VS530FL	TCCCGGATCCGCGACAGCTTTATTGTTTAG	This study	BamHI
VS531RS	ATTGAGATCTTGTTTTGATATTATACCATTC	This study	BglII
VS531RL	TATCAGATCTGAAATTCCTTTCAAGAATTAAATTG	This study	BglII
VS534FS	CGTAGGATCCTATATTAAAATTATACAATATC	This study	BamHI
VS534F	TATTGGATCCATCGCTCTTGATCCCTTCAGTG	This study	BamHI
VS534R2	AAATAGATCTATTAAAACTTTTTTAAATCG	This study	BglII
VS535FS	TTTTGGATCCTATCTCCTAATTATAG	This study	BamHI
VS535F	TAGGGGATCCTTCACAATAGCATACCTAAAG	This study	BamHI
VS535R	AAAAAGATCTCTTATGATTCGTTCAAAAATTTC	This study	BglII
VS536FS	ATCTGGATCCCACAAATCCATTATATAG	This study	BamHI
VS536FL	GGTTGGATCCTTTTGGTTTTTAAAGAAG	This study	BamHI
Lux537R2	TTATAGATCTAAATATCAATACATTTTACC	[27]	BglII
VS537RL	TTGCAAAGATCTTATAGTTTTTGTAACC	This study	BglII
VS543FS	CAAGGGATCCTATTTATCTATGATACTATG	This study	BamHI
VS543F	TTTGGGATCCTTTAATACTCCTCTATTTGTTG	This study	BamHI
VS543R	TCACAGATCTTTTGGCTTGCCCTATTGCTG	This study	BglII
VS546FS	ATTGGGATCCATCGCTTGAGTATATC	This study	BamHI
VS546F	AAAAGGATCCGCGTTTCCTTTCAAATTGAAATC	This study	BamHI
VS546R	TCTAGGATCCTGCTTAAAATGGAGCTTTATTC	This study	BamHI
VSOrfXFS	TTCTGGATCCAAATTCGTTCATTTTAG	This study	BamHI
VSOrfXFL	TGTTGGATCCGTGAATCACAAACGCTTAATTG	This study	BamHI
VS547RS	CATGAGATCTAACATTACCATTATACCAC	This study	BglII
VS547RL	CGTTAGATCTTGTTTCTCCTTACTATAC	This study	BglII
Lux546F	TATAGGATCCTATATACTTTATGGTAAGC	This study	-
Lux547R	TATAGATCTACCTAGTTTCATACCTATC	This study	-

a Restriction site added for cloning purposes are underlined.

### Generation of P*cag-lux* and P*cag-5′UTR-lux* reporter strains

*H. pylori* G27-derivative strains carrying the transcriptional fusions of the P*cag* promoter regions (with or without the 5′ untranslated regions) with the *luxCDABE* reporter operon were obtained as described previously [27]. Briefly, the P*cag* promoter regions were PCR amplified from *H. pylori* G27 genomic DNA, digested and cloned into the unique BamHI site of the vector pVCC. Constructs carrying the insert in the desired orientation were identified by digestion and checked by sequencing, then the plasmids were used to transform G27*lux* acceptor strain carrying the promoterless *Photorhabdus luminescens luxCDABE* operon in the *vacA* locus. The chloramphenicol-selected mutant strains were expanded and the correct insertion was confirmed by PCR. pVCC-derivative constructs used for *H. pylori* transformation and the corresponding mutant strains are reported in Table 1.

### RNA preparation

To measure metal-dependant transcriptional responses, cultures of the wild-type and mutant strains were grown to mid-log or late-log phase (optical density at 600 nm [OD_600_] 0.5–0.6 and 1.7, respectively) and treated for 30 minutes with either 1 mM (NH_4_)_2_Fe(SO_4_)_2_, 1 mM NiSO_4_, or 100 µM 2,2-dipyridyl chelator (Sigma-Aldrich) prior to RNA extraction. For acid exposure experiment, mid-log cultures (OD_600_ 0.5–0.6) of the wild-type and mutant strains were divided in two subcultures and treated with either 1 M HCl to adjust the pH from 7.0 to 5.2 (acid shock) or equal volume of sterile water (control sample). Subcultures were grown for 30–90 minutes before RNA extraction. The volume of 1 M HCl required to achieve a pH of 5.2 was determined on aliquots of the growing cultures. To follow the expression of *cag* genes over time, an overnight culture of wild type strain was diluted to a starting OD_600_ of 0.08 and cultured to an OD_600_ of 1.75 for approximately 15 hours. Aliquots of 10 ml from this master culture were harvested at different time points for RNA extraction. Heat-shock experiments were performed as previously described [28]. Total RNA was extracted using a hot-phenol procedure [23]. RNA integrity and purity were ensured by electrophoresis on 1% agarose gels.

### Primer extensions

Primer extension analyses were performed with 12 µg of total RNA and 0.1 pmol of 5′-end-labeled primers as described previously [29]. The oligonucleotides used for primer extension reactions are listed in Table 2. Quantification of the signals from extension products obtained was performed using a Storm phosphor-imager (Amersham-GE) and Image Quant Software (Molecular Dynamics).

### Overexpression and purification of recombinant His_6_-Fur

Recombinant His_6_-Fur was overexpressed and purified under native conditions [26]. Thrombin protease (10 U/mg) was used to remove the N-terminal histidine tag according to the instructions of the manufacturer (Amersham GE Healthcare). The purified, untagged protein was dialyzed overnight against binding buffer (10 mM Tris-Cl, pH 7.85, 50 mM NaCl, 10 mM KCl, 0.02% Igepal CA-630, 10% glycerol, 0.1 mM dithiothreitol). A Bradford colorimetric assay kit (Bio-Rad) was used to quantify the protein fractions with bovine serum albumin as standard.

### DNase I footprinting

Plasmid pGEM-P*cagAB* used for the generation of the footprinting DNA was obtained by cloning the 403 bp Lux547R-Lux546F amplicon in pGEM-T Easy (Promega). 1 pmol of pGEM-P*cagAB* was linearized with NcoI, dephosphorylated with calf intestinal phosphatase and labeled at the 5′ ends with [γ-^32^P]ATP (6,000 Ci/mmol; PerkinElmer) and T4 polynucleotide kinase (all enzymes by New England Biolabs). The labeled DNA probe was further digested with SalI and the products were separated by native polyacrylamide gel electrophoresis and purified as described previously [29]. The binding reactions between approximately 20 fmol of labeled probe and increasing concentrations of Fur were carried out at room temperature for 15 min in a final volume of 50 µl in footprinting buffer (10 mM Tris-Cl, pH 7.85, 50 mM NaCl, 10 mM KCl, 0.02% Igepal CA-630, 10% glycerol, 5 mM dithiothreitol) containing either 150 µM (NH_4_)_2_Fe(SO_4_)_2_ or 150 µM 2,2-dipyridyl, with 300 ng of salmon sperm DNA (Invitrogen) as a nonspecific competitor. Afterwards, DNase I (0.08 U), diluted in footprinting buffer containing 10 mM CaCl_2_ and 5 mM MgCl_2_ was added to the reaction mixture and digestion was allowed to occur for 85 s. The reaction was then stopped, and the samples were extracted and purified [26]. Samples were resuspended in 5 µl of formamide loading buffer, denatured at 100°C for 3 min, separated on 8 M urea-6% acrylamide sequencing gels and autoradiographed.

### AGS cell culture and infection assay

AGS cells, a human adenocarcinoma epithelial cell line (ATCC CRL 1739), were grown in RPMI-1650 medium with 10% fetal bovine serum (FBS) in tissue-culture flasks. For the infection assay, cells were seeded in 24-well plates (Orange Scientific) and cultured for 1–2 days to reach 60–80% confluence. Before the infection, the wasted medium was replaced with fresh RPMI-1650 with 5% FBS conditioned in the bacterial incubator (9% CO_2_, 91% air atmosphere, and 95% humidity). Cells were infected with G27 P*cag-lux* strains at a multiplicity of infection (MOI) of 5, while other 24-well plates filled with medium but without AGS cells were infected with the same amount of bacterial culture and used as control sample. The plates were placed inside the bacterial incubator and luminescence was measured at regular time intervals with Victor3V (1420) multilabel reader (Perkin Elmer), with bottom trail pre heated at 37°C. Luminescence was measured with an integration time of 2 seconds (normal aperture) in the absence of optical filters. The luminescence values of wells filled with plain growth medium were used as blank control and subtracted from the values of the experimental samples. Each infection assay was performed in quadruplicate and the assay was repeated in four independent biological replicates. average values and standard deviations were calculated.

## Results and Discussion

### Mapping of *cag* promoters

**Figure 1:**
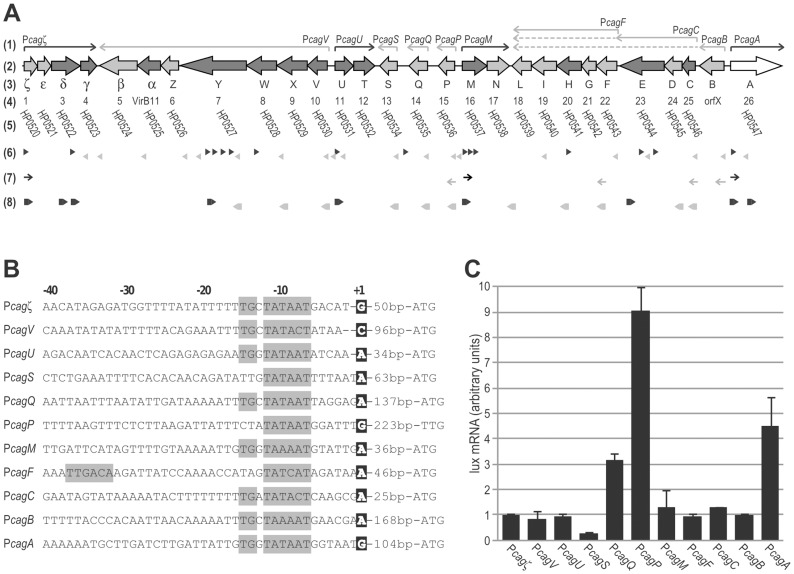
Mapping of *cag* promoters. **A**. Genomic organization of the *cag* pathogenicity island in the *H. pylori* G27 strain. (1) Transcriptional units mapped in this study; black: plus strand; grey: minus strand. (2) Annotated *cag* genes and ORFs; white block arrow: ORF encoding effector toxin CagA; dark grey block arrows: ORFs encoding structural T4SS components; light grey block arrows: ORFs encoding components with ancillary or unknown function. (3) Alphabetic classification of *cag* genes. (4) Numeric classification of *cag* genes. (5) Annotation of *cag* genes in reference strain 26695. (6) Transcriptional start sites mapped in [20]. (7) Transcriptional units identified in [17], [33]. (8) Transcriptional units identified by unbiased promoter-trapping experiments in [30] **B**. Summary of relevant features within the nucleotide sequences of the P*cag* promoters mapped in this study. The TSSs (+1) are boxed in black boxes and showed in boldface. Sequences corresponding to -10 regions, the extended TGn elements and recognizable -35 region are enlightened in grey boxes. **C**. Comparison of the transcript levels at the P*cag* promoters fused with *lux* reporter genes. mRNA levels of the P*cag*-*lux* constructs were assayed by primer extension analysis using the oligonucleotide VSluxC1 and quantified with a phospoimager. The mean values from three independent experiments are reported as arbitrary units of ^32^P counts.

Recent studies have provided insights on the transcriptional organization of the *H. pylori cag* pathogenicity island, with the mapping of transcriptional start sites (TSS) and the identification of putative promoter regions. In strain 26695, out of 40 putative 5′-end of RNA transcripts identified [20], 14 map within the 300 bp upstream of annotated ORFs, and are predicted to contain the promoter regions of Cag protein coding sequences. These results were recently confirmed in different strains by promoter-trap and reverse transcription analyses of ORFs and intergenic regions [30]. The positions of the 5′ end of RNA transcripts and transcriptional units identified are schematically represented reported in Fig. 1A. To study their regulation we set out to map the 5′ end of these transcripts by primer extension analyses on total RNA extracted from *H. pylori* strain G27 grown to mid-log phase using oligonucleotides mapping downstream of the 14 aforementioned predicted promoters (Fig. 1A, Table 2). The remaining 26 internal and antisense TSSs deserve more dedicated studies and have been deliberately excluded from the current study.

Primer extension results mapped 11 out of 14 predicted TSSs (Fig. 1B), suggesting that the *cag*-PAI of strain G27 harbors at least 11 transcriptional units: *cagζεδγ*, *cagVXW*YZ*αβ*, *cagU*T, *cagS*, *cagQ*, *cagP*, *cagM*N, *cagFGHIL*, *cagCDE*, *cagB* and *cagA* (Fig. 1A). By contrast, we were unable to detect TSSs located upstream *cagγ*, *cag*Z and *cagW* genes, reported by [30], possibly due to strain-specific differences in the nucleotide sequences of the promoters, or due to transcript levels below the sensitivity of our technique.

To evaluate the transcript levels at the *cag* promoters and compare their relative mRNA abundance, we used *H. pylori* G27 isogenic strains harboring the transcriptional fusions of the *cag* promoters with a *lux* reporter system [27]. For each of the 11 *cag* promoters, the region upstream of the transcriptional start site was placed upstream of the promoterless *lux* reporter operon, generating 11 P*cag-lux* strains (Table 1). Total RNA was extracted from the P*cag*-*lux* strains grown to mid log phase and used to quantify transcript levels by primer extensions with a common lux specific primer, with data normalized to the mRNA level at the P*cagζ* promoter (Fig. 1C). For the sake of clarity we will synthesize the results by subdividing the *cag* genes/operons in three different functional classes: i) *structural cag genes* or operons coding for components forming the structural core of the secretion system, essential for CagA translocation and IL-8 induction (dark grey block arrows in Fig. 1A); ii) *effector cag genes* coding for secreted components responsible for cytotoxic effects on host cells, such as CagA (white block arrow); and iii) *accessory cag genes* or operons, which may have modulatory or ancillary functions (light grey block arrows).

Interestingly, the promoters of operons encoding structural Cag components (P*cagζ*, P*cagV*, P*cagU*, P*cagM*, P*cagF*, P*cagC*), essential for a functionally assembled *cag*-T4SS, are all characterized by a reasonably conserved -10 box in addition to an extended TG element or -35 box (Fig. 1B). These promoters display similar basal transcription levels, suggesting that the messenger RNAs of these structural operons are generated with similar stoichiometric ratios. On the other hand, the P*cagA* promoter responsible for the expression of the CagA toxin, has a perfect match to the -10 TATAAT box in addition to an extended TGn, and a putative UP element [17], which together appear to confer significantly higher transcription levels with respect to the operons encoding structural components (Fig. 1C). This observation is congruent with the different (effector) role for the *cagA* gene product, in accordance with previous observations [30]. The promoters of monocistronic operons encoding putative accessory components (P*cagS*, P*cagQ*, P*cagP*, P*cagB*), not essential for T4SS function, exhibit conserved -10 boxes, but lack extra elements. Interestingly, they denote extremely disparate basal transcription levels, with P*cagQ* and *PcagP* matching or even exceeding the high basal levels of *cagA* transcripts (intriguingly P*cagP* lacks conserved -35 or TG elements, tentatively indicating at a dedicated activator involved in the high transcription levels). This suggests that the genes under the control of these promoters are actively expressed, and likely play an important functions for the *cag* secretion system.

These transcript levels verified in the G27 strain, show some differences compared to the *cag* transcript levels reported in other *H. pylori* strains [20], [30]–[32]. In particular, P*cagC* and P*cagζ* were previously reported with a 10- to 1000-fold higher expression level with respect to the other structural promoters in the C57 and 26695 strains [33], [34], suggesting strain-specific transcriptional variations.

### Growth-phase regulation of the *cag* promoters

**Figure 2:**
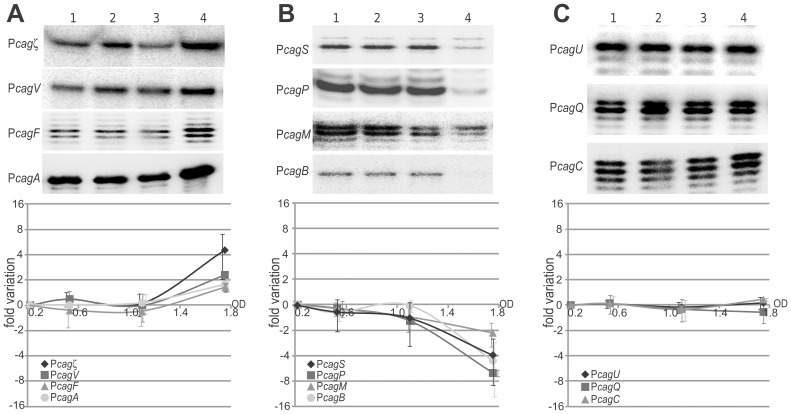
Growth phase-dependent regulation of *cag* promoters. The P*cag* promoters are reported according to the variations of the transcript levels during bacterial growth, with promoters induced at late logarithmic phase (A), repressed during bacterial growth (B) and not sensitive to growth phase-dependent (C). An overnight culture of wild type strain was diluted to an OD_600_ of 0.08 and cultured for 15 hours. Total RNAs were extracted from equal volumes of cultures at different time points corresponding to OD_600_ of 0.22 (t1), 0.53(t2), 1.06(t3) and 1.75(t4). Results from the primer extension analysis are shown in the upper panels. The normalized average intensities of the bands from three independent experiments are reported in the graphs as the n-fold change, with error bars indicating the standard deviation.

To study the transcriptional regulation of the selected *cag* promoters during growth, we carried out time course experiments. Aliquots of bacterial cultures were collected at different time points and used to extract total RNA for quantitative primer extension experiments at the 11 *cag* promoters (Fig 2).

Transcription from the P*cagζ*, P*cagV*, P*cagF* and P*cagA* promoters showed no significant variation in the amount of mRNA during the early exponential growth stages of the bacteria, while their amount increased in late logarithmic growth phase with an up to five-fold increase of transcript levels from P*cagζ*, P*cagV*, P*cagF* and P*cagA* (Fig. 2A). By contrast, transcription from P*cagS*, P*cagP*, P*cagM* and P*cagB* promoters exhibited a progressive reduction of mRNA levels during the time course experiment, reaching up to ten-fold down-regulation at OD = 1.7 (Fig 2B). No significant variations of transcripts deriving from P*cagU*, P*cagQ* and P*cagC* were recorded during the same time-course experiment (Fig. 2C).

We conclude that during bacterial growth, transcription from P*cagζ*, P*cagV*, P*cagF* and P*cagA* promoters increases at late log-phase, in agreement with previous observations [32], [35], [36], while transcription from P*cagS*, P*cagP*, P*cagM* and P*cagB* promoters is decreased. These observations suggest that the transition from exponential to stationary phase prompts a modulation in the expression of the CagA toxin and of specific structural components of the T4SS, which together may impact on the assembly or function of the secretion system. In other pathogenic bacteria, such as *Brucella abortus* and *Legionella pneumophila*, the regulation of essential components of the T4SS is growth-phase dependent [37] or quorum-sensing responsive [38]. Thereby, the virulence mechanisms are fine-tuned according to the bacterial load and the nutrient availability in the host niche. The growth-phase dependent regulation of particular *cag* promoters indicate that *H. pylori* may adopt similar strategies to control virulence, as *cag*-specific responses to stress signals encountered in the host niche after infection are frequently mimicked by the stationary phase conditions of planktonic laboratory cultures [31].

### Environmental regulation at the *cag* promoters

**Figure 3:**
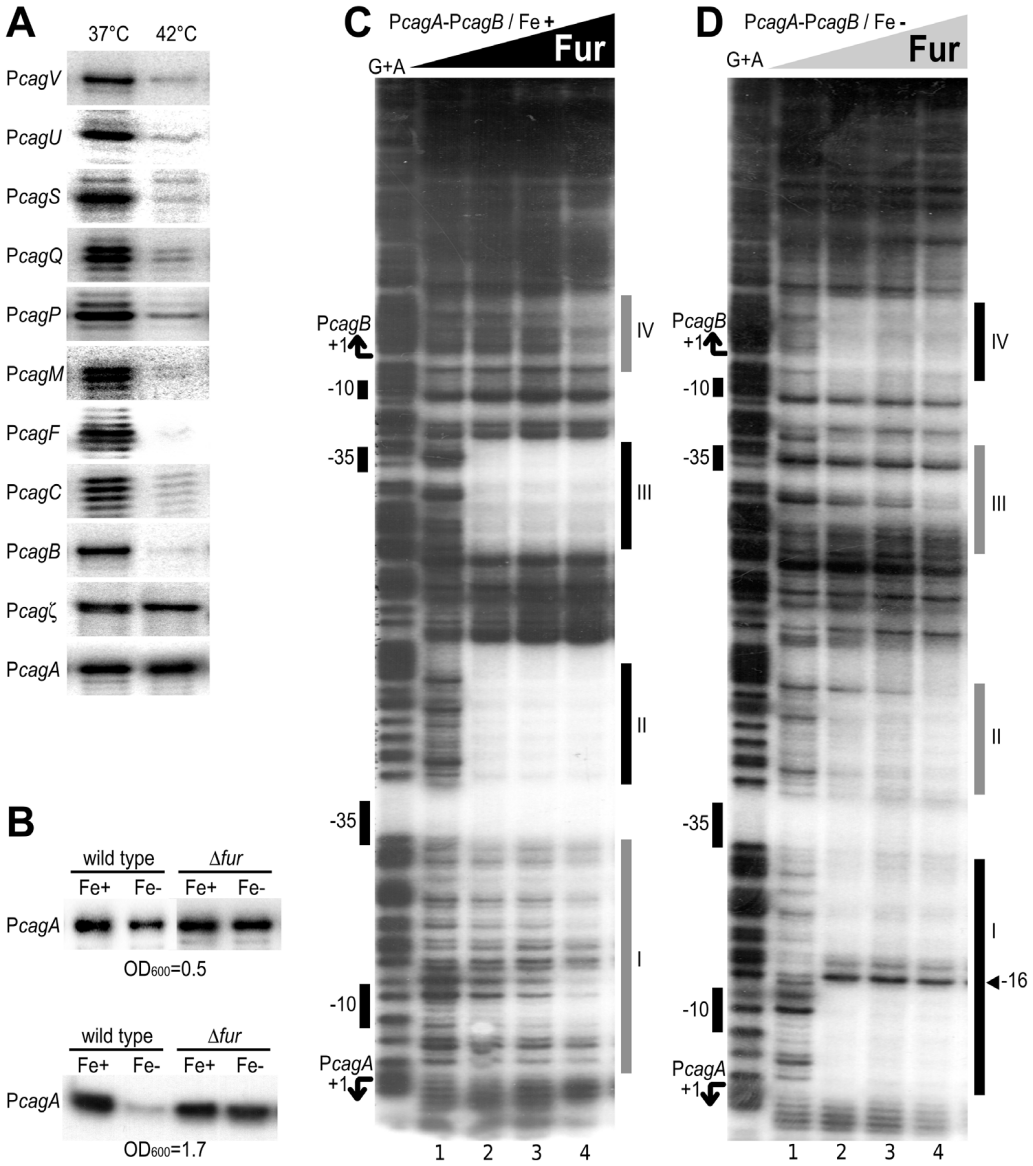
Stress responses of *cag* promoters. **A**. Heat-shock response of the P*cag* promoters. Primer extension analyses were performed on total RNA extracted from bacterial cultures of *H. pylori* wild type strain grown to exponentially phase and maintained at 37°C or exposed to 42°C for 30 min. **B**. Iron-dependent regulation of the P*cagA* promoter. Liquid cultures of wild type and Δ*fur* strains were grown to OD_600_ = 0.5 or OD_600_ = 1.7 and treated for 30 min with 1 mM (NH_4_)_2_Fe(SO_4_)_2_ (Fe+), or 100 µM 2,2-dipyridyl (Fe-). mRNA levels at the P*cagA* promoter were assayed by quantitative primer extension on the total RNA extracted. **C**. In vitro binding of Fur protein to the P*cagB*-P*cagA* promoter region in a DNaseI footprinting assay. Concentrations of Fur dimer, 0 nM (lane 1), 21 nM (lane 2), 42 nM (lane 3), 84 nM (lane 4), 168 nM (lane 5) and 336 nM (lane 6). The binding reaction was performed in a final volume of 50 µL in presence of divalent iron ions as cofactors (150 µM (NH4)_2_Fe(SO_4_)_2_). The vertical grey and black bars on the right of the panel (I-IV) indicate the areas of partial and complete DNaseI protection, respectively, resulting from binding of Fur on the probe. Fur binds to nucleotide positions +2 to -14 (I), -20 to -63 (II), -108 to -115 (III) and -145 to -188 (IV) with respect to the +1 transcriptional start site (TSS) of P*cagA*. An hypersensitivity band that appears at high concentrations of Fur is indicated by a black triangle. On the left side of the panel, the TSS downstream P*cagB* and P*cagA* are indicated with bent arrows, while the relative position of the -10 and -35 regions of the 2 promoters are indicated as vertical black boxes. G+A: G+A sequencing reaction ladder. **D**. DNaseI footprinting of Fur protein to the P*cagB*-P*cagA* promoter region without the supplement of iron ions and with 150 µM 2,2-dipyridyl used to sequester the Fe^2+^ ions. The experimental conditions used for the footprinting assay are the same as described in panel C.

To study the transcriptional regulation of *cag* promoters in response to environmental changes, we exposed exponentially growing cultures of *H. pylori* G27 strain to various stress conditions that challenge the bacterial metabolism or fitness. Total RNA was extracted from treated and untreated samples and transcript levels at the *cag* promoters were assayed by quantitative primer extensions with *cag*-specific oligonucleotides (Table 2). In bacterial cultures exposed to heat shock (30 min at 42°C) we observed a 6- to 40-fold reduction of mRNA levels at most *cag* promoters (Fig. 3A). Exceptions to this finding were at the P*cagζ* and P*cagA* promoters that showed unchanged transcript levels (Fig. 3A). Subsequently, we assayed the mRNA levels at all *cag* promoters in *H. pylori* strains deleted of the heat shock transcriptional regulatory genes *hspR* and *hrcA* [39]. In comparison to the wild type strain, the knock-out Δ*hspR* and Δ*hrcA* mutants grown in normal conditions or exposed to heat shock treatment showed similar mRNA levels at *cag* promoters (data not shown). Thus, the observed variation in the mRNA levels after heat shock is not under the direct control of HspR or HrcA, likely reflecting a pleiotropic effect on transcription or mRNA stability.

Bacterial cultures treated with iron (30 min with 1 mM (NH_4_)_2_Fe(SO_4_)_2_), iron chelator (30 min with 100 µM 2,2-dipyridyl), or nickel (30 min with 1 mM NiCl_2_), showed no significant variations in the transcript levels from most of the *cag* promoters (data not shown). Exception to this finding was observed at the P*cagA* promoter that showed a slight increase in the RNA levels after exposure to iron ions and 1.5-fold reduced levels in iron-depleted cultures (Fig. 3B – upper panel). This iron-dependent response is in agreement with previous studies [22].

### Fur-dependent iron-inducibility of *cagA*

To further investigate on iron-dependent regulation, we assayed the mRNA levels at the *cag* promoters in the knock-out mutant of the iron-dependent regulator Fur, exposed to the same conditions as the wild type strain. In RNA extracted from the mutant culture strain we observed unchanged transcript levels at the P*cagA* promoter (Fig. 3B – middle panel), as well as at the other *cag* promoters (data not shown). The loss of the iron-dependent response of the P*cagA* promoter in the Δ*fur* mutant strain suggested that Fur can mediate the iron–dependent regulation at this promoter.

Since intracellular Fur increases during bacterial growth [23], we assayed the iron-dependent response of the P*cagA* promoter in wild type and Δ*fur* cultures grown to late log-phase (OD_600_ = 1.7), with results reported in Fig. 3B (bottom panel). As expected, in the wild type background the P*cagA* promoter was iron-regulated, with markedly higher differences in the mRNA levels between iron-replete and iron-depleted conditions, while in the Δ*fur* strain, the transcript levels were unchanged. These results suggested that Fur represses the P*cagA* promoter in response to iron starvation, likely through a direct mechanism. Albeit related indications were previously reported [21], [22], [40], together with extensive Fur binding within the *cag*-PAI [23], the mechanism behind the iron-inducibility of *cagA* had not been understood in detail so far.

Thus, to demonstrate direct Fur-promoter interaction and map its operators sites, we set up DNaseI footprinting assays using the P*cagA-*P*cagB* intergenic region as probe, both in iron-replete (*holo*-Fur) and iron-depleted (*apo*-Fur) conditions. The protection pattern of *holo*-Fur on the P*cagA*-P*cagB* probe (Fig. 3C) shows four areas of DNaseI protection (marked I-IV in Fig. 3C): two high affinity binding sites (*holo*-operators; II and III) appear at the minimal protein concentration used (21 nM, lane 2), while two low affinity binding sites (I and IV) appear a higher Fur concentrations (84 nM, lane 4). Footprinting of the *apo*-Fur on the same probe showed a swap of the protection affinities, with two high affinity binding regions (*apo*-operators I and IV, Fig. 3D) and two lower affinity binding regions (II and III). Both the *apo*-operators and the *holo*-operators respectively encompass sequences similar to the TCATT-n10-TT and TAATAATnATTATTA consensus motifs recently proposed for discriminative *apo*- and *holo*-Fur binding [40]-[42]. Given the position of the *apo*-Fur binding sites with respect to the *cagA* transcriptional start site and the Fur-dependent iron-response in stationary phase cultures, we propose that the P*cagA* promoter is directly regulated by an *apo*-Fur repression mechanism involving the occlusion of the -10 box to the RNA polymerase. In fact, the position of these boxes suggest a repressor role exerted by *apo*-Fur, at least on P*cagA*, in agreement with the *apo*-Fur-dependent repression of its transcript (Fig. 3B). This mechanism is similar to the FeON regulation mechanism described for the *pfr* promoter [26], [42], and likely important for the repression of the CagA toxin when the intracellular concentration of Fe^2+^ ions are limiting. Moreover, the position of the high affinity binding site of *holo*-Fur upstream the -35 region of the P*cagA* promoter may indicate that the *holo*-form of the protein could positively regulate the P*cagA* promoter, with a class II activation mechanism. Hence, the Fur-mediated regulation of P*cagA* is dependent by a complex binding of *holo*- and *apo*-Fur proteins on the corresponding operators. Recent studies suggest that iron limitation may increase *cag*-dependent virulence [43]. Thus it is possible that additional regulatory mechanism concur to regulate the functionality of the *cag*-T4SS. For example, previous footprinting analysis with the α-subunit of the RNA polymerase showed a protection pattern on the region spanning from -17 to -70 nucleotides of P*cagA* [17], suggesting the presence of an UP element recognized by the CTD domain of RpoA. We can speculate that the observed iron-dependent regulation of P*cagA* could be exerted not only by the binding of *apo*- and *holo*-Fur to its operator elements, but also by Fur competing for the binding of theα-subunit to the UP element. These evidences add to the documented importance of Fur as central regulatory hub in the *H. pylori* pathogenesis.

### Acidic shock response of *cag* promoters

**Figure 4:**
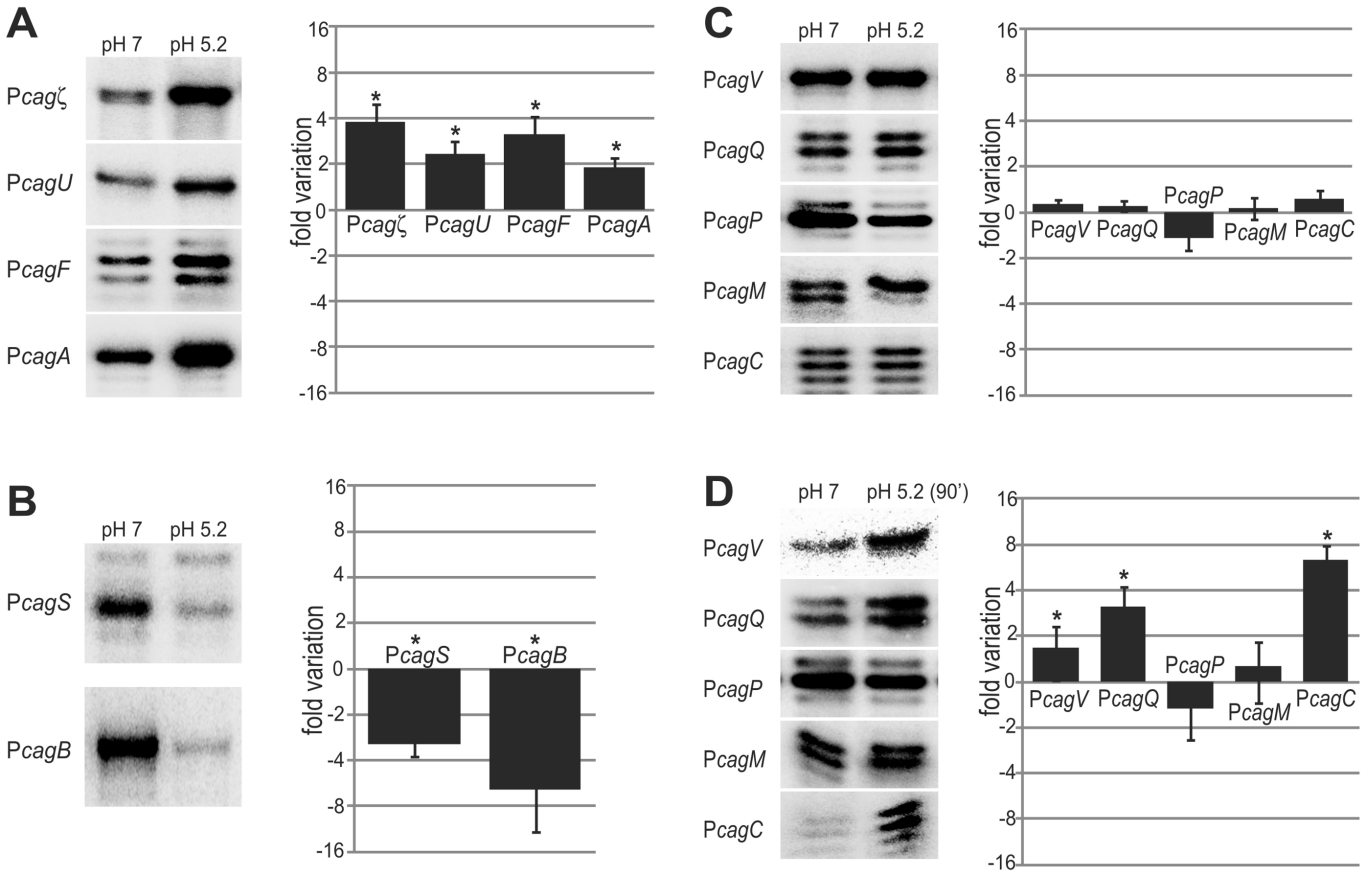
pH-dependent response of *cag* promoters. Primer extension analyses were performed on total RNA extracted from bacterial cultures of *H. pylori* wild type strain, grown to exponential phase and treated for 30 or 90 min with 22 mM HCl to adjust the pH of the medium to 5.2, or maintained at neutral pH (pH 7.0). The intensity of the bands of four independent experiments were quantified and reported in the graphs as n-fold variation of the transcript levels in the acidic-treated samples with respect to the untreated sample. **A**. P*cag* promoters with transcript levels increased after an acidic treatment for 30 min; **B**. Promoters with reduced mRNA levels after the 30 min acidic treatment; **C**. Promoters with unchanged transcript levels after the treatment. Error bars indicate the standard deviation and significant variations between treated and untreated samples are marked with asterisks (*P*<0.05). **D**. Response of P*cagV*, P*cagQ*, P*cagP*, P*cagM* and P*cagC* promoters after an acidic treatment of 90 min.

To investigate the transcriptional responses to acidic pH, liquid cultures of *H. pylori* grown to mid-log phase were divided in two subcultures and treated for 30 min or 90 min either with HCl to adjust the pH of the medium to a value of 5.2 (acid shock) or with the same volume of sterile water (untreated sample). The RNAs extracted from three independent cultures were assayed by primer extension experiments and bands were quantified with results reported in Fig. 4.

Upon 30 min acidic treatment, transcript levels from the P*cagζ*, P*cagU*, P*cagF* and P*cagA* promoters increased a 3.8-, 2.3-, 3.2- and 1.9-fold, respectively (Fig. 4A). In contrast, transcript levels from the P*cagS* and P*cagB* promoters decreased 3.1- and 6.2-fold, respectively (Fig. 4B), while no significant variation in the mRNA levels was observed at the P*cagV*, P*cagQ*, P*cagP*, P*cagM* and P*cagC* promoters (Fig. 4C). After a 90 min acidic shock treatment, most of the promoters showed a pattern of RNA accumulation similar to the 30 min treatment (data not shown). Exceptions were observed at the P*cagV*, P*cagQ* and P*cagC* promoters for which the mRNA level increased to 1.7-, 3.2-, 6.3-fold, respectively (Fig. 4D). Thus, almost all operons coding for proteins essential for the formation of a functional T4SS are inducible by low pH, including P*cagA*, P*cagV* (core), P*cagU* (core), P*cagC* (pilum), P*cagF* (pilum stabilization), P*cagζ* (transglycosylase and core stabilization). These results are in agreement with previous studies, showing pleiotropic responses of the *cag* promoters to acidic stress in different *H. pylori* strains [18], [19], [44], [45]. One exception is represented by *cagM*, expressing a gene product involved in the stabilization of the T4SS core, whose transcript levels appear unchanged upon acidic treatment. On the other hand, the operons coding for unessential, ancillary *cag* components (P*cagS*, P*cagQ*, P*cagP*, P*cagB*) exhibited distinct responses, with P*cagS* and P*cagB* being repressed by acidic treatment. Interestingly, these promoters respond to acid treatment, and are co-regulated in the stationary phase of growth, tentatively pointing at a common function, which deserves to be further investigated in the future.

**Figure 5:**
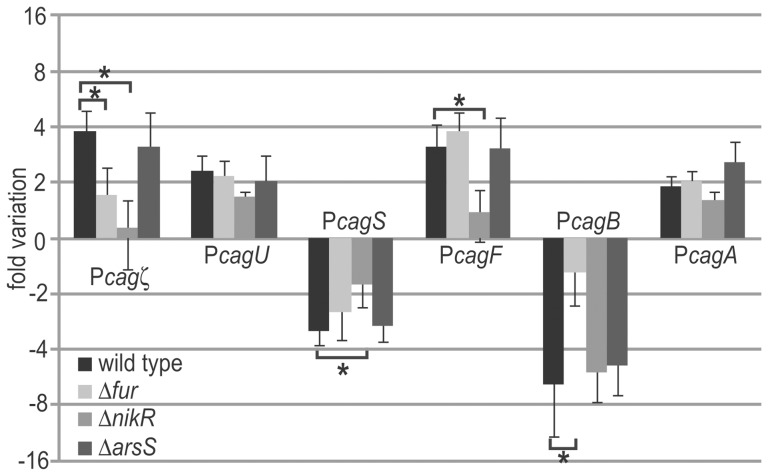
Acid-dependent response of *cag* promoters in Δ*fur*, Δ*nikR* and Δ*arsS* mutant strains. Cultures were grown to exponential phase and exposed to acid-shock (pH = 5.2) for 30 min. Transcript levels at the P*cagζ*, P*cagU*, P*cagF*, P*cagA*, P*cagS* and P*cagB* promoters were assayed by quantitative primer extensions. Asterisks mark the significant differences of n-fold variations deriving from the average band intensity of three independent primer extension experiments. Error bars indicate the standard deviation.

As the acidic-response in *H. pylori* is primarily controlled by the ArsRS two-component system, together with the metal responsive transcriptional regulators NikR and Fur [2], [24], [44], [46], we cultured wild type, Δ*fur*, Δ*nikR* and Δ*arsS* strains to mid-log phase, exposed to acidic shock for 30 min and evaluated the mRNA levels at the acid-responsive P*cagζ*, P*cagU*, P*cagF*, P*cagA*, P*cagS* and P*cagB* promoters (Fig. 4 A and B) by quantitative primer extension assays with results reported in Fig. 5. Intriguingly, P*cagF* and P*cagS* promoters showed a loss of the pH-inducible response in the Δ*nikR* mutant, displaying unchanged transcript levels after acidic treatment with respect to the untreated sample, while in the Δ*fur* and Δ*arsS* mutants an acid response similar to the wild type strain was observed. Likely, the acidic response at these promoters is directly or indirectly mediated by NikR. Similarly, transcript levels at the P*cagB* promoter were unchanged after acidic treatment in the Δ*fur* mutant, while the wild type strain and the other mutants showed a pH-induced reduction in the mRNA levels. These results suggest that Fur is involved in the acid-dependent repression of P*cagB*. On the other hand, P*cagζ* appears to loose the pH-inducible response both in *fur* and *nikR* knockout strains, suggesting a role for both regulators on its acidic regulation. Finally, variations of transcript levels in the mutant strains similar to that in the wild type strain were observed at the P*cagA* and P*cagU* promoters, hence acid response of these promoters is mediated by still unknown factors.

To further investigate on the observed loss of the acidic response of some P*cag* promoters in the mutant strains, we performed footprinting assays of recombinant Fur protein on a labeled probe encompassing the P*cagζ* promoter, while footprinting assays of recombinant NikR were performed on the DNA fragments corresponding to the P*cagζ*, P*cagS*, P*cagF* promoters. No patterns of DNaseI protection were observed on these probes (data not shown), suggesting that NikR and Fur mediate the acid responses at these promoters indirectly.

### P*cag* response to bacterium-host contact

**Figure 6:**
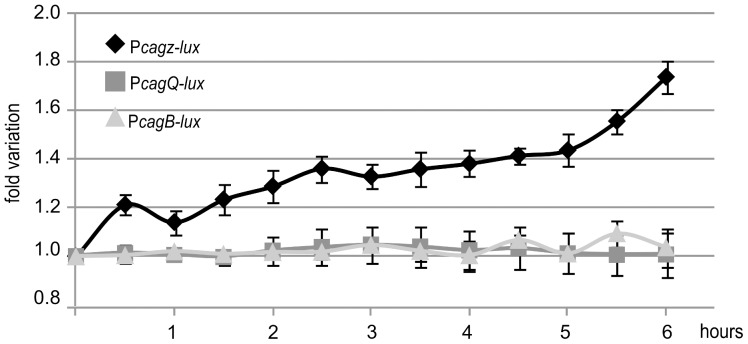
Reporter assays with the P*cag-lux* strains in host cell co-cultures. Liquid cultures of P*cagζ-*, P*cagQ-* and P*cagB-lux* strains were added at a multiplicity of infection of 5 to 24-wells plates containing human gastric adenocarcinoma (AGS) cells or with the medium only. The luminescence emitted by the reporter strains was recorded by a multilabel reader at regular intervals. Signals were normalized on the samples without AGS cells and averaged. Standard errors were calculated from four independent experiments (each in four technical replicates).

Host cell contacts are potent elicitors of secretion system gene expression in pathogenic bacteria [15]. To assess the possible *in vivo* effects exerted by bacterium-host contacts on the transcription of the *cag* promoters, we used co-cultures of AGS cells and *H. pylori* G27-derived strains carrying the P*cag-lux* transcriptional fusions. Bacterial cultures were grown to mid-log phase and used to infect AGS cells cultured in 24-well plates (AGS^+^ sample), while same amounts of bacterial cultures were added to plates containing only the medium (AGS^-^ sample). During a time-course experiment, we measured the luminescence of the samples at regular time intervals, and for each time point we calculated the ratio of the signal from the bacteria grown in presence or absence of AGS cells (AGS^+^/AGS^-^ ratio). The P*cagζ*-*lux* strain exhibited a significant increase of luminescence when co-cultured in the presence of AGS cells, with an AGS^+^/AGS^-^ ratio that increased over time (Fig. 6). In contrast, the other P*cag-lux* strains showed no significant differences between samples cultured with or without the AGS cells, with an AGS^+^/AGS^-^ ratio unchanged during the experiment, as exemplified by the P*cagQ-* and P*cagB-lux* strains (Fig. 6). We can conclude that under the experimental conditions tested, the interaction of *H. pylori* with its host cells exerts a positive transcriptional effect only on expression levels at the P*cagζ* promoter. Previous studies indicated that contact of *H. pylori* with host cells provokes the increase of visible T4SS pili extruding from the bacterium at the host-pathogen interaction surface [47], and that the protein composition of the *cag*-T4SS pili differs if bacteria are grown planktonically or in co-culture with AGS host cells [48]. The finding that the interaction with host cells rapidly induces the transcription of the P*cagζ* promoter is, therefore, particularly striking. In fact, the *cagζεδγ* operon encompasses *cagδ*, a gene that codes for a factor bridging the periplasm across the inner and outer membrane, essential for the stabilization of the T4SS core, as well as *cagγ*, encoding the transglycosylase involved in the local hydrolyzation of the murein layer important for the formation and extrusion of the assembling T4SS. Interestingly, Kim and colleagues reported similar variations of *cagδ* expression in *H. pylori* 69a strain, co-cultured with AGS cells for 1 hour [25]. These evidences suggest a conserved regulation of the operon, likely due to the modulation of the P*cagζ* promoter activity. Together, the results indicate that P*cagζ* induction may modulate the number of pili, their distribution on the bacterial cell surface and their composition after host cell contact. Previous observations of the AGS-induced regulation of other *cag* promoters (e.g. *cagA*, *cagP* and *cagS*) [20], [49], were not confirmed in this study, possibly due to strain-specific responses to host-cell contact, or due to the different reporter system used to monitor the responses.

### Post-transcriptional regulation

**Figure 7:**
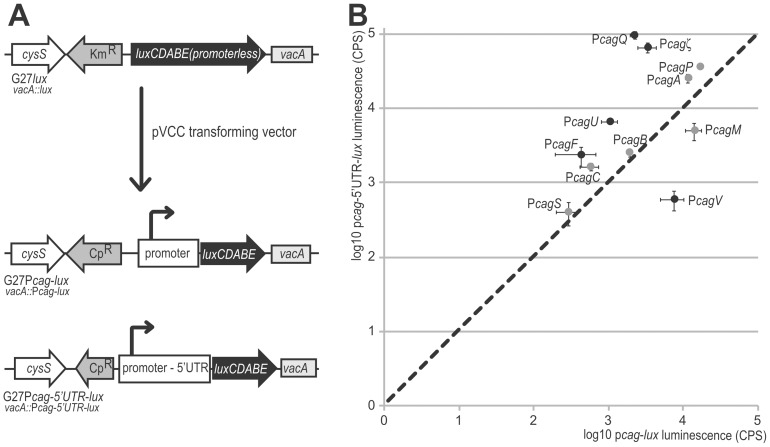
Comparison of the P*cag*-*lux* and P*cag*-5′UTR-*lux* reporter signals. **A**. Schematic representation of the P*cag*-*lux* and P*cag*-5′UTR-*lux* fusion constructs, obtained transforming the G27*lux* acceptor strain with the PVCC vector. The promoter sequences with or without the 5′untranslated regions (5′UTRs) carried by the pVCC vector are inserted upstream the *luxCDABE* operon by double homologous recombination and selected by *cat* chloramphenicol resistance. **B**. Luminescence signals from three independent experiments were normalized according to the optical density of the cultures and the means values were reported in the graph, with P*cag*-*lux* signals on the X-axis and P*cag*-5′UTR-*lux* signals on the Y-axis. Error bars indicate the standard deviation. A dashed line was added to the graph, corresponding to the 1∶1 ratio of the two signals. Grey dots: *cag* promoters with 1∶1 signal ratio; black dots: *cag* promoters with altered P*cag*-*lux/*P*cag*-5′UTR-*lux* signal ratio.

The analyses of the sequences downstream the transcriptional start sites of the P*cag* promoters showed that *cag* transcripts harbor 5′ untranslated regions (5′UTRs) of different lengths (Fig. 1B). To assess possible post transcriptional effects mediated by the P*cag* 5′UTRs, in analogy to similar findings reported in *A. tumefaciens* [50], we used the aforementioned P*cag*-*lux* reporter fusions and *ad hoc* P*cag*-5′UTR-*lux* constructs encompassing also the 5'UTRs downstream of the promoters (Fig. 7A). The luminescence emitted by mid-log growing cultures of these reporter constructs was compared to the corresponding P*cag*-*lux* constructs without the 5′UTR region. Except for P*cagζ*, P*cagM, PcagF* and P*cagC*, the luminescence counts of the 5'UTR-less constructs correlated well with the transcript levels assayed in primer extension analysis (Fig.1C; 7/11 promoters matching). We observed a nearly 1∶1 signal ratio between the constructs with or without the 5′UTR for P*cagA*, P*cagB*, P*cagC, PcagM, PcagS* and *PcagP* (Fig. 7B), suggesting that most 5′UTRs downstream of *cag* promoters do not affect the stability or the translational efficiency of the nascent messenger RNAs. Intriguingly, the luminescence of P*cagV-*5′UTR*-lux* constructs decreased significantly with respect to the 5′UTR-less construct (Fig. 7B), suggesting that this sequences could contain elements that reduce the translational efficiency or decrease the mRNA abundance.

On the other hand the P*cagζ*-, P*cagU-*, P*cagQ*-, P*cagF-*5′UTR-*lux* constructs showed a strong increase of luminescence with respect to the corresponding 5′UTR-less constructs (Fig. 7B), suggesting that the 5′UTR downstream of these promoters could contain elements that enhance mRNA stability or translation.

## Conclusions

In conclusion, the general picture emerging for the *cag*-T4SS regulation can be synthesized as follows: i) despite the lack of dedicated transcriptional regulators encoded within the PAI, the *cag* cistrons appear to be subjected to a complex network of direct and indirect regulations; ii) operons coding for structural components of the T4SS display homogeneous transcript levels; they are transcriptionally responsive to growth-phase, and indirectly responsive to pH and other stress-factors. In some cases (P*cagV*, P*cagQ*, P*cagζ* and to lesser extent P*cagU, PcagF*) they are subjected to post-transcriptional control; iii) only P*cagζ* transcription appears to be triggered immediately after host-cell contact; iv) the *cagA* effector gene is highly transcribed, and matches the responses to acidic pH and bacterial growth phase together with other co-regulated operons encoding structural *cag* components. On the contrary, it is not responsive to heat stress as most of the other *cag* operons, while it is clearly induced by iron in a direct *apo-*Fur-dependent regulation mechanism; iv) monocistronic operons encoding accessory factors vary considerably in their basal transcription levels and responses, indicating non-constitutive expression of their components which may be involved in physiologically relevant aspects of *cag*-T4SS maturation and assembly. For example, previous reports indicate that *cagP* products may be involved in *H. pylori* adherence to host-cells [51]. The finding that *cagP* exhibits the highest transcript levels of the *cag*-PAI, and that it is down-regulated in stationary phase, when the transcription levels of structural and effector *cag* operons increase, provides an exemplification on how the dissection of *cag* transcriptional responses may guide our efforts to understand the *cag*-T4SS function. This knowledge will have important outcomes for the appropriate management of *H. pylori* infections, as the *cag*-T4SS is among the most important pathogenetic factors that carry an increased risk for gastric cancer [52].
